# Extraction and Nano-Sized Delivery Systems for Phlorotannins to Improve Its Bioavailability and Bioactivity

**DOI:** 10.3390/md19110625

**Published:** 2021-11-05

**Authors:** Tianjian Tong, Xiaoyang Liu, Chenxu Yu

**Affiliations:** 1 National Engineering Research Center for Seafood, Dalian Polytechnic University, Dalian 116034, China; 2 Department of Agricultural and Biosystems Engineering, Iowa State University, Ames, IA 50011, USA; tianjian@iastate.edu

**Keywords:** phlorotannins, nano delivery systems, bioavailability, bioactivity

## Abstract

This review aims to provide an informative summary of studies on extraction and nanoencapsulation of phlorotannins to improve their bioavailability and bioactivity. The origin, structure, and different types of phlorotannins were briefly discussed, and the extraction/purification/characterization methods for phlorotannins were reviewed, with a focus on techniques to improve the bioactivities and bioavailability of phlorotannins via nano-sized delivery systems. Phlorotannins are promising natural polyphenol compounds that have displayed high bioactivities in several areas: anticancer, anti-inflammation, anti-HIV, antidiabetic, and antioxidant. This review aims to provide a useful reference for researchers working on developing better utilization strategies for phlorotannins as pharmaceuticals, therapeuticals, and functional food supplements.

## 1. Introduction

Phlorotannins are a large group of chemical compounds found in brown algae (kelp, rockweeds, etc.), accounting for about 5–12% of the dry mass [1]. Phlorotannins have attracted substantial attention from the research community due to their high bioactivities [2,3,4,5,6,7,8,9,10,11,12], which could be utilized in various medical and nutritional enhancement applications [5,6]. Phlorotannins are natural compounds with low toxicity compared to man-made compounds [13,14], which has added to their appeal. 

Phlorotannins are tannins exist primarily in brown algae [1,15]. Phlorotannin content in seaweeds can also vary for individual species, geographic regions, and extraction techniques [16]. They are developed from phloroglucinol (1,3,5-trihydroxybenzene) via acetate-malonate (polyketide) pathway which are different from other hydrolysable and condensed tannins [14,17], with molecular weight ranging from 126 to 1 × 100 KDa [18,19,20,21,22]. The structural differences among phlorotannins are widely recognized. There are six different types (i.e., classes) of phlorotannins: fucols, fucophlorethols, fuhalols, carmalols, phlorethols, and eckols, as listed in Table 1 [19,20,21,22,23]. Inside each class, phloroglucinol (1,3,5-trihydroxybenzene) monomers can bind to one another at different locations of the ring of phloroglucinol [17]. These free bindings lead to all kinds of isomer structures in addition to the conformational ones [17]. From another perspective, we can re-group phlorotannins into two basic groups: linear phlorotannin and branched phlorotannin [17], with linear phlorotannin having two terminal phloroglucinol monomer residues and branched phlorotannin having three terminal phloroglucinol monomer residues. 

The composition, amount, and types of phlorotannins obtained from seaweed such as brown algae could be dependent on the extraction techniques used. Traditionally, phlorotannin are isolated via organic solvent extraction followed by chromatographic techniques to purify the compounds [24,25,26]. More recently, hot-pressurized liquid extraction (HPLE) [2,27,28,29,30,31,32] and integrated process of HPLE-resin purification (HPLE-RP) have also been developed [33,34], which produced higher phenolic compound yields than traditional methods. Typical liquid extraction (LE) involves using one polar solvent (water in most cases) and one non-polar solvent [35,36,37], and phlorotannins transfer from the polar solvent to the non-polar one with chemical potential being the driving force of this transfer [35,36,37]. Most conventional LE methods have in common “cons” of being “non-green” due to usage of organic solvents [34]. In a recent effort, in which boiling water was used instead of “non-green” solvent as the extraction solvent, Chowdhury et al. [38] reported a recovery yield of dieckol from *E. cava*, *E. stolonifera*, and *E. bicyclis* to be 86%, 93%, and 98%, respectively. More detailed discussion on extraction methods can be found in Section 2 below.

Phlorotannins have been shown to have anti-inflammatory, anti-HIV, anticancer, antidiabetic, and antioxidant functionalities. Phlorotannins are effective regulators of several biochemical processes linked to the disruption of homeostasis in major chronic diseases [18,39]. Inflammation is part of the process by which the immune system defends the body from harmful agents, such as bacteria and viruses [40,41,42,43]. Phlorotannins isolated from different brown algae demonstrated anti-inflammatory bioactivity in many cases: phlorotannin isolated from *E. cava* can suppress oxidative stress and inflammatory mediators [44], and phlorotannin isolated from *E. stolonifera* can suppress iNOS and expression of COX-2 gene [45], to name a few examples. Phlorotannin was also reported to have anti-HIV activities [11]. 6,6′-bieckol isolated from *E. cava* can suppress HIV-1-induced syncytia formation and production of viral p24 antigen [11]. Phlorotannins were also shown have anticancer bioactivity [12,46]: eckol can suppress tumor growth in vivo on S180 xenograft-bearing mice [46]. They were also reported to have antidiabetic qualities [47,48,49,50,51,52,53,54] by suppressing α-glucosidase, PTP 1B, aldose reductase, ACE, AGEs, and aldose reductase [47,48,49,50,51,52,53,54]. To be more specific, dioxinodehydroeckol can suppress α-glucosidase and PTP 1B [47]; 7-phloroeckol can suppress α-glucosidase and PTP 1B [47]; dieckol can suppress α-glucosidase [53]; and 7-phloroeckol can suppress ACE and α-glucosidase [53]. 

Last by not least, phlorotannins are strong natural antioxidants [55,56,57,58,59,60]. Dieckol can suppress UV-B radiation induced photo-oxidative stress in human fibroblast cell line and reduce cell damage [55]; diphlorethohydroxycarmalol (DPHC) isolated from *I. okamurae* can scavenge UV-B radiation induced ROS in human fibroblast cells [57]; and dieckol can reduce cell damage induced by UV-B radiation both in vitro (HaCaT cells) [61] and in vivo (zebra fish) [59]. Phlorotannins can also potentially be used as functional ingredients in skin care products to offer protection against photo-induced skin damages.

**Figure 1:**
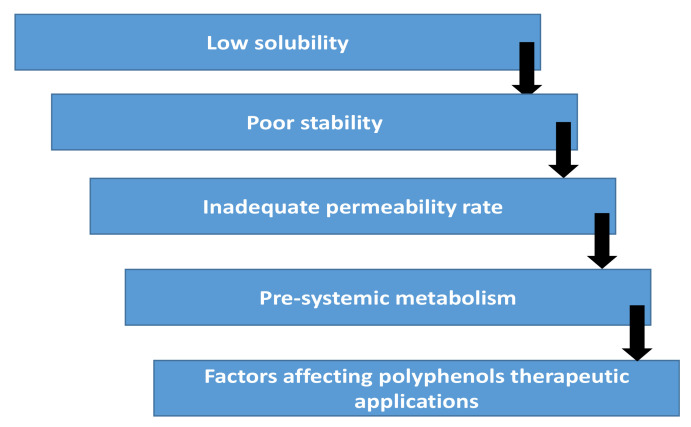
Factors affecting polyphenols therapeutic applications, replicated from Conte et al. [65] with permission.

**Figure 2:**
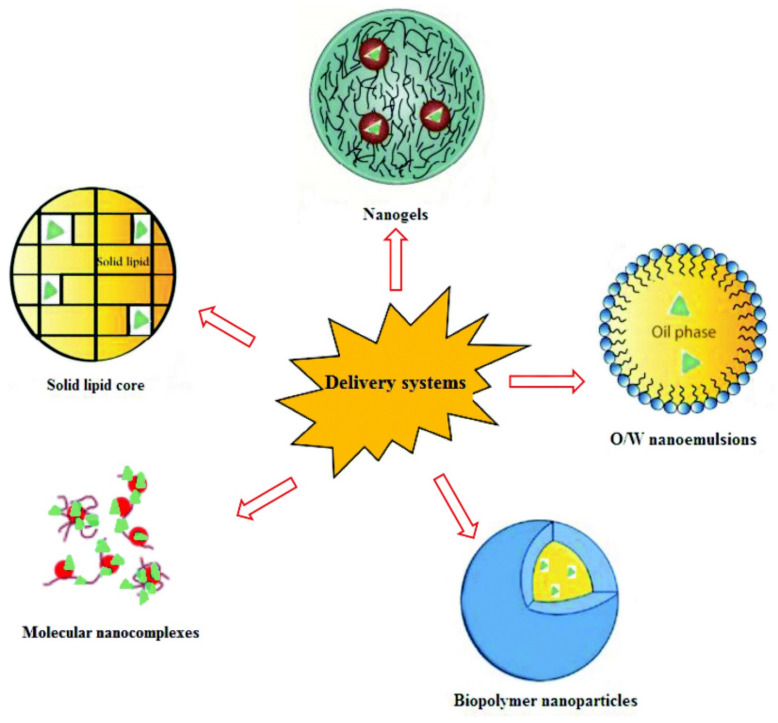
Schematic representation of nano-sized delivery systems, replicated from Qi et al. [71] with permission.

Although phlorotannins have many attractive biofunctionalities, as members of the polyphenol family, phlorotannins also suffer from low bioavailability, which limits their utilization. Polyphenols’ low bioavailability is mainly due to their low absorption in the human gastrointestinal (GI) tract following consumption and their extensive biotransformation within the gut, which may lead to their rapid clearance from the body (Figure 1 [62]). To improve the bioavailability of phlorotannins, nano-sized delivery systems have been investigated [26,61,63,64,65,66,67,68,69,70]. Figure 2 showed a schematic representation of examples of these systems [71]. These nano-enabled approaches could protect phlorotannins against transformation in the gut; they can also improve delivery/absorption of phlorotannins [61]. Nano-phlorotannin systems were also shown to enhance the functionality of the phlorotannins. For example, gold nanoparticle–phlorotannin and silver nanoparticle–phlorotannin complexes have been investigated by serval researchers [26,61,63,64,65,66,67,68,69,70]; they were shown by DPPH assays to have elevated antioxidant activity. Nano-enabled improvement on bioavailability and bioactivity may offer a more effective way for the utilization of phlorotannins in pharmaceutical, food, and biochemical industries.

## 2. Extraction and Purification of Phlorotannins

The operation of phlorotannin extraction aims to achieve two major goals: one is to extract as high a quantity of phlorotannins, primarily from brown algae, as possible; another is to maintain high bio-integrity and bioactivities of the phlorotannins [7,8,9,10,11,12]. These two goals could be in conflict: phlorotannins are unstable polyhydroxylated molecules, and they can easily be oxidized [17]. Considering the different types of phlorotannins and their susceptibility to various physical/chemical processing, development of different isolation/extraction methods suitable for different types with specific biofunctionality [17] remains a challenge [18,19]. A quick summary of the pros and cons of common isolation/extraction methods reported by different research groups follows.

HPLE methods: HPLE is generally considered environmentally friendly to an extent, if water is used instead of organic solvents [2,27,29,31,32]. Low cost is another advantage of HPLE [28,30]. HPLE can also be scaled up to a certain extent to go beyond lab scale, but its yield as of today is still not high [29]. Another restriction of HPLE is that it requires the solvent to be kept in liquid state during the entire extraction process, which can be an operational challenge [29]. In addition, macroalgae might contain heavy metal [72], which could be extracted together with phlorotannins by HPLE [72] and become enriched. 

HPLE-RP methods: to reduce the risk of heavy metal being enriched along with phlorotannins, resin purification (RP) methods were introduced [33,34]. With RP, heavy metal content in the phlorotannins extracted from macroalgae can be dramatically reduced [33] Kim et al. [33] compared four types of resins: HP-20, SP-580, XAD-7HP, and XAD-2. All four types of resins displayed effective purification of phlorotannins [33]. Among them, HP-20 resins displayed the highest absorption and desorption capacities, and this led to the best purification performance. 

Liquid chromatography (LC) is another widely utilized [13,25,45,55,73,74,75,76] separation method to isolate phlorotannins from algae extracts. It was used for phlorotannins from *E. cava* [55], *E. stolonifera* [45], fucales [13], *F. vesiculosus* [25,75,76], and fucus of the northern portuguese coastline [76]. It was also used for isolating and purifying pyrogallol-phlroglucinol and phlorofucofuroeckol from brown algae [45,74]. Although HPLC works really well in laboratories, it is not suitable for large scale industrial production.

Liquid–liquid extraction (LLE) of phlorotannin: LLE is a separating method in which one polar and one non-polar solutions are used together [35,36,37,77]. Phlorotannins transfer from one liquid (polar) to another (non-polar). Chemical potential is the driving force of this transfer [35,36,37,77]. LLE is a commonly utilized separating method [35,36,37,77]. However, it had its own limitations in separating polarity-related compounds [35,36,37,77].

One-step centrifugal partition chromatography (CPC) method: Most liquid extraction methods mentioned above have common “cons”, which are that “non-green” solvents are used during the extraction process [34]. A novel method was introduced in a recent study, in which a quick, one-step CPC (centrifugal partition chromatography) system was utilized to isolate four types of phlorotannins (dieckol, phlorofucofuroeckol-A, 7-phloroglucinol-6,6-bieckol, and pyrogallol-phloroglucinol-6,6-bieckol) from *E. cava* [78]. HPLC analyses indicated that all four types of phlorotannins, dieckol, phlorotucofuroeckol-A, 7-phloroglucinol-6,6-bieckol, and pyrogallol-phloroglucinol-6,6-bieckol, were extracted at 90% yield [79]. Compared to traditional approaches with long and complicated processes resulting in high losses of phlorotannins [8,80], the loss of phlorotannins in one-step CPC was much reduced [78]. 

NEDES extraction: Another new development in phlorotannin extraction was the use of deep eutectic solvents (DESs). DES is a new class of ionic liquid (IL), typically formed by mixing choline chloride with hydrogen bond donors [81]. Environmentally friendly natural DESs (NADESs) were first explored for extracting active compounds from plant raw material [82,83]. Oblichinskaya et al. reported the first effort of using NADES to extract phrolotannins from brown algae *F. vesiculosus* L. and *A. nodosum* (L.) Le Jolis [84]. Ten NADES with different compositions were studied for their efficiency of extracting phlorotannins. They found that aqueous solutions of NADES based on choline chloride and lactic acid were the most efficient, comparable to those of traditional extractants such as Me_2_CO_3_ and EtOH and almost 10 times more efficient than pure NADES. As NADESs are clean green solvents, they could become an alternative to organic solvents to reduce the environmental footprint of phlorotannin extraction.

Following extraction, purification and characterization are the next steps. Molecular size-based separation is often used to purify different types of phlorotannins: these methods usually involve dialysis or ultrafiltration (UF) or a combination of the two [85]. Dialysis is an approach to isolate phlorotannins by different molecular weight [79,86,87]. The proper selection of membrane pore size is key to separating high molecular weight (MW) phlorotannins from low MW ones [79,86,87]. As for the UF system, with a fixed flow rate, UF can have a better performance [85]. The capacity of the UF system reported in the literature is still limited to lab-scale operations, ranging from 100 mL to 10 L [85]. One advantage of the UF system is its wide range of MW cutoff (MWCO), but industrial-ready UF for phlorotannins remains to be developed for large-scale applications [85].

After purification, NMR spectroscopy, mass spectrometry, and advanced chromatography are three common tools to evaluate the phlorotannin product to characterize the level of polymerization and the molecule weight distribution [88]. Phlorotannins could be indirectly quantified via the Folin-Ciocalteu (F-C) method, which measured the antioxidant activities in a sample. Although the F-C method is not selective for phlorotannins, it could yield quick readings with a simple operation, short turnaround time, and high throughput [20,79,89,90,91]; hence, it has been used for rapid determination of phlorotannin content in brown algae extracts [92]. In addition, computational chemistry was another useful tool [93,94,95]. Computational chemistry method can provide a cost-efficient supplementary tool to NMR spectroscopy [93,94,95,96]. Density functional theory (DFT) quantum chemical calculation was introduced to determine electronic structure of phlorotannins [96]. Natural bond orbital (NBO) was introduced to determine the donor acceptor stabilization energy caused by intramolecular and intermolecular hydrogen bonds in phlorotannins [96]. 

## 3. Bioactivities of Phlorotannins

As a group of tannins, phlorotannins from brown algae were shown to be effective in regulating several biochemical processes linked to the disruption of homeostasis in major chronic diseases [18,39]. They have been revealed to have a range of diverse biological functions and activities, such as antibacterial [7], anti-inflammatory [8], antioxidant [9], antidiabetic [10], anti-HIV [11], and anticancer [12,97,98] activities. They can form complexes with pro-oxidant proteins, chelate metal ions, or directly trap reactive oxygen species (ROS) to modulate cellular responses to stresses and/or injuries [99]. The most studied bioactivity of phlorotannins is their ability to scavenge radicals due to donation of their hydrogen atoms or electrons [100,101,102,103,104,105], which can be evaluated via 2, 2-diphenyl-1-picrylhydrazyl (DPPH) radical-scavenging assay [88,106,107], ferric reducing/antioxidant power (FRAP) assay [108], dichloro-dihydro-fluorescein diacetate (DCFH-DA) assay [109], trolox equivalent antioxidant capacity (TEAC) assay [110], and electron spin resonance spectroscopy [107].

Table 2 listed some of reported anti-inflammation effects of phlorotannins from literature. This list is by no means comprehensive, but it showcases the great potential of phlorotannins as anti-inflammation agents. 

In addition, phlorotannins isolated from brown algae have also been widely reported to have anticancer bioactivities. For example, eckol from brown algae was shown to suppress tumor growth in vivo in S180 xenograft-bearing mice [46].

An intriguing aspect of phlorotannins is their anti-HIV activities. As listed in Table 3, phloroglucinol compounds from territorial plants (e.g., *M. japonicus*) and synthesized dimeric phloroglucinol were shown to be promising as part of a treatment plan against AIDS. 6,6’-bieckol isolated from *E. cava* was also shown to be effective. Other phlorotannin species should be further explored in future research.

Phlorotannins have also been demonstrated to be effective against diabetes [51,52,53,54,55,56,57,58]. These antidiabetic bioactivities included suppressing α-glucosidase, phosphotyrosine phosphatase 1B (PTP 1B), aldose reductase, angiotensin-converting enzyme (ACE), and advanced glycation end products (AGEs) [51,52,53,54,55,56,57,58]. Table 4 listed some of the reported work on phlorotannins as potential antidiabetes treatments

Last but not least, phlorotannins, as with other polyphenols, are good natural antioxidants that could bring health benefits to consumers [102,103,104,105,111,116]. ROS, hydrogen peroxide (H_2_O_2_), hydroxyl radicals (HO·), and superoxide anions can all be produced during oxygen metabolism [60,102,103,104,105,117,118,119], which could cause oxidative stress to cells that can lead to cell damages and cancerogenic mutations. Phlorotannins can scavenge these free radical species to remove them from harm’s way. In addition, phlorotannins can act as total antioxidant with high reducing power. Free radical scavenging of phlorotannins extracted from *S. aquifolium* could be as high as 6.770 ± 0.001 mg phlorotannin g^−1^ dry weight (DW), 6.1290 ± 0.0200 mg ascorbic acid g^−1^ DW, 19.7210 ± 0.0300 mg FeSO_4_ g^−1^ DW, and 76.28 ± 0.20% of 25 mg DPPH mL^−1^ extract [120]. Since higher amounts of phlorotannin content correlated to higher antioxidant activity [9,121], better delivery of phlorotannins to improve their bioavailability would lead to elevated bioactivities as well. 

When compared to other organs, skin was more vulnerable to UV (UV-A and UV-B radiation) radiation [122,123,124]. Exposure to long-term UV radiation can lead to skin cancers, photoaging, and even suppression of immune system [123,125,126,127]. Phlorotannins were shown to be effective in reducing photo-induced oxidative stress and ameliorating radiation damage to skins. In Table 5 below, different types of phlorotannins’ antioxidant bioactivities are summarized.

In addition to these bioactivities, phlorotannins were also reported to have antibacterial activities [128], antiadipogenic activity to ameliorate obesity [129,130], antivirus [116,131,132], anti-Alzheimer’s disease [133], anti-arthritis [134] and antiallergic activities [111,135,136,137]. In summary, phlorotannins, as a group of highly active polyphenols, have great potential for applications in pharmaceutical, cosmetic, health care, and food industries.

## 4. Nano-Sized Delivery Systems for Phlorotannins to Enhance Bioavailability and Bioactivities

Although phlorotannins have all the highly desirable bioactivities and functionalities summarized in Section 3, their utilization as pharmaceuticals, therapeuticals, and food supplements is often limited due to their low bioavailability. Phlorotannins are unstable in the acidic condition of the gut and the alkaline condition of the small intestine; if not protected, they could easily be transformed and/or metabolized [138]. Phlorotannins are water soluble, but their absorption in the human gastrointestinal (GI) tract following consumption could be low, as evidence suggested that phlorotannins were mainly absorbed after metabolized in the large intestine [139], not in the stomach and the small intestine. A number of strategies have been used to increase the chemical stability of phlorotannins and to improve their transportation/absorption in the GI tract via nano-sized delivery systems that involved the encapsulation of phlorotannins in nanovectors, which offer protection against chemical/enzymatic transformation. These nano-systems could improve the distribution and overall bioavailability and bioactivity of the phlorotannins [65]. Such systems differ for the internal structures (e.g., core–shell-like vs. embedding matrixes) and the physical/chemical states of the encapsulated active ingredients (i.e., the phlorotannins), and their encapsulation efficiencies (EE, defined as the experimental loading/theoretical loading × 100%) were different. Effective drug delivery systems usually require an EE to be higher than 60%, which could be used as a reference to evaluate various phlorotannin nano-delivery systems. 

Nano-sized delivery systems utilize nanoparticles or nanostructures with diameters ranging from 1–1000 nm to encapsulate/carry phlorotannins as the “payload”. These nanoparticles, due to their size, exhibit properties and phenomena attributable to their dimension [140]. Nano-sized encapsulation systems can be made via several different approaches based on chemical, physical, and physiochemical principles. Chemical encapsulation (e.g., interfacial and in situ polymerization methods) usually requires the polymerization of monomers that cross-link with the payload [141]; physical encapsulation, on the other hand, usually uses already formed polymers (and natural polymers) to form a matrix in which the payload can be entrapped via various methods including air suspension, pan coating, spray drying, spray congealing, and micro-orifice systems [65]. Physiochemical methods (e.g., coacervation, phase separation, complex emulsion, meltable dispersion, and nanoprecipitation) aim to form stable nano-sized payload-carrying systems through particle size reduction processes [142]. Nanoencapsulation techniques, especially physical and physiochemical methods, have been [140] widely investigated for improving bioavailability and bioactivities of polyphenols by protecting the polyphenols against digestive transformation/degradation, reducing their toxicity and improving their water solubility [65]. Various nanocarrier systems, including cyclodextrins, nanospheres, nanocapsules, solid lipid nanoparticles, liposomes, and micelles, have been explored for polyphenols in general. Here, we will review a few specific cases in which phlorotannins were the payloads.

Shibata et al. [71] studied phlorotannins extracted from brown algae *E. bicyclis*, *E. cava*, and *E. kurome*; they showed that, with a liposome carrying system, the nanoencapsulated phlorotannins showed potent inhibition of phospholipid peroxidation at 1 μM, had significant radical scavenging activities against the superoxide anion (50% effective concentration values: 6.5–8.4 μM) and DPPH (50% effective concentration values: 12–26 μM), and were more effective compared to ascorbic acid and α-tocopherol. Furthermore, a nanocarrier system of phlorotannins and soybean protein prepared by co-extrusion was shown to have a pronounced DPPH radical scavenging activity. The authors concluded that the nanoencapsulated phlorotannins could be a good functional food or supplement with anti-inflammatory activity.

**Figure 3:**
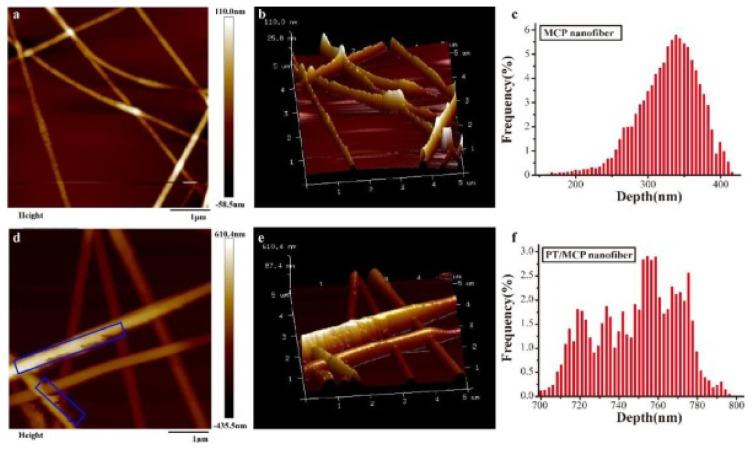
Surface, three-dimensional AFM images, and depth distribution of MCP nanofibers (**a**–**c**) and PT/MCP nanofibers (**d**–**f**). Replicated from Cui et al. [67] with permission.

Physical encapsulation was also applied to phrolotannins. In a recent effort, Cuong [69] reported nano phlorotannin powder made by spray-drying of phlorotannins isolated from *S. serratum* grown in Vietnam. The optimum spray drying condition was established as follows: a carrier-to-solution ratio of 10%, compressed air pressure of 0.8 bar, liquid feed speed of 10 mL min^−1^, and inlet temperature of 110 °C. Under these conditions, the antioxidant activity of phlorotannin powder possessed total antioxidant activity at 4.347 ± 0.018 g ascorbic acid equivalent 100 g^−1^ DP, reducing power activity at 9.390 ± 0.024 g FeSO_4_ equivalent 100 g^−1^ DP and DPPH free radical scavenging activity at 70.02 ± 0.26%. The phlorotannin content and antioxidant activities of the nano-sized powder particles were affected by the spray drying condition (*p* < 0.05). These particles were nano-sized with morphology of irregular shapes. These nano phlorotannin powders displayed antioxidant activities for potential application in pharmaceuticals and nutritional supplements. In another effort [81], electrospun polycaprolactone (PCL)/phlorotannin micro/nanofibres containing different phlorotannin (from *E. cava*) concentrations (1, 3, and 5 wt%) were fabricated. Due to their hydrophilicity and water absorption ability, phlorotannin-containing fibrous mats exhibited outstanding wettability compared with pure PCL fibrous mats. The biocompatibility of the mats was examined in vitro using osteoblast-like cells (MG63). The phlorotannins were shown to promote alkaline phosphatase (ALP) activity and calcium deposition, which could induce bone regeneration. Cell viability (at 5 wt% phlorotannin), total protein content, ALP activity, and calcium deposition were all higher with PCL/phlorotannin mats than with pure PCL mats. These results suggest that electrospun nano PCL/phlorotannin fiber is a promising bioactive material for enhancing bone tissue growth. Lin et al. also demonstrated phlorotannin-encapsulating nanofibers with blended sodium alginate (SA) and poly(ethyleneoxide) (PEO) as the matrix via electrospinning, The optimum blending ratio to produce quality nanofibers was found to be 50:50:10 (SA/PEO/Ph); the nanofibers were shown to have an average diameter of 331 nm. The fibers were then used as an antibacterial reagent against *S. enteritidis* on chicken at 4 and 25 °C with great performance; the cell count drastically decreased from 6.20 to 3.28 Log CFU/g at 4 °C and decreased from 8.80 to 2.53 Log CFU/g at 25 °C. Their application significantly increased the shelf-life of chicken, which suggested that these nanofibers could be good food packaging materials due to the activities of the phlorotannins. Cui et al. [67] used a natural polymer (*Momordica charantia* polysaccharide (MCP)) as a nanofiber matrix to encapsulate phlorotannins via cold plasma treatment. The successfulness of PT loading into MCP nanofibers was confirmed using SEM, AFM, TGA, and DSC, as shown in Figure 3. After cold plasma treatment, the release efficiency of PT from the nanofibers was enhanced by 23.5% (4 °C) and 25% (25 °C). In addition, both antioxidant and antibacterial activities of the MCP–phlorotannin nanofibers were markedly improved. These nanofibers could be a novel functional food packaging material. In this paper [67], the authors reported that both MCP and phlorotannin exhibit antidiabetic and antioxidant properties. They demonstrated that an MCP nanofiber–phlorotannin complex can enhance antioxidant effects. Thus, this MCP–phlorotannin complex was also used to make novel and enhanced food packaging materials.

**Figure 4:**
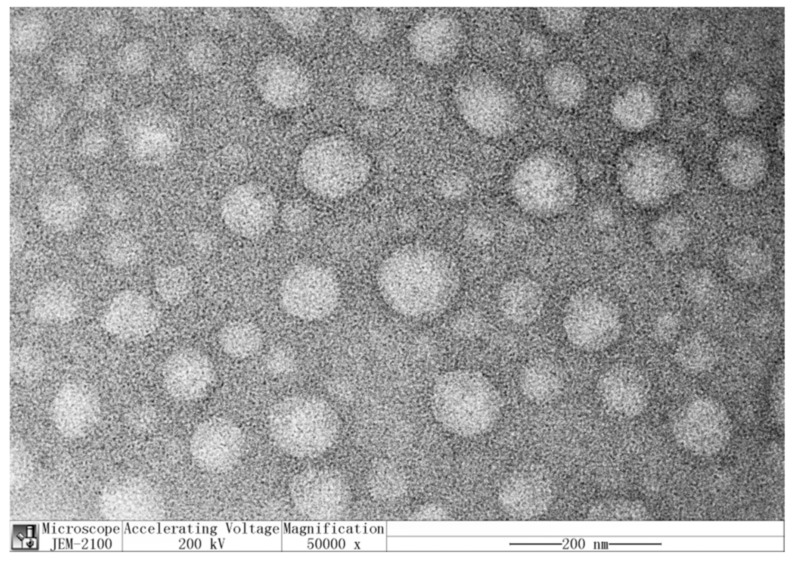
Transmission electron microscopy (TEM) image of phlorotannin@PVP nanoparticles (PPNPS). The concentration of PPNPS was 1 mg/mL, replicated from Qi et al. [66] with permission.

**Figure 5:**
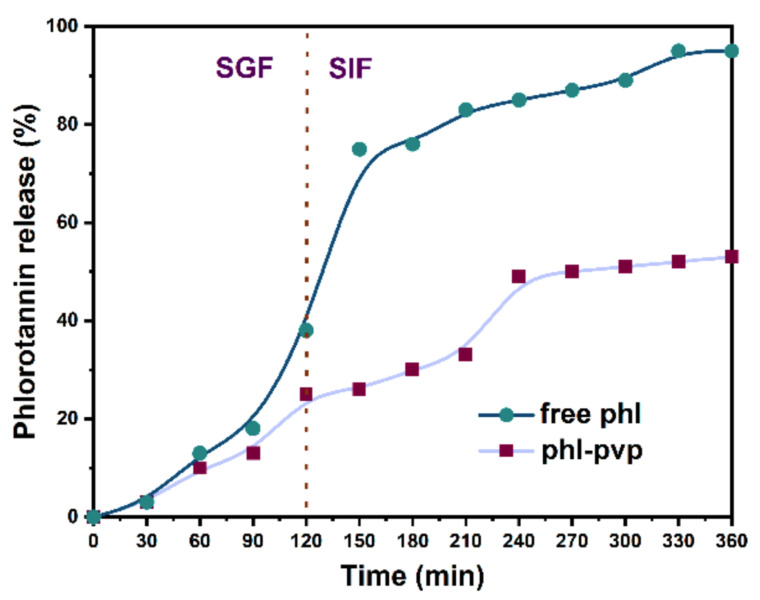
Release profile of free phlorotannin and Phlorotannin@PVP nanoparticles (PPNPS) (1:8, *w/w*) in simulated gastrointestinal fluids, replicated from Qi et al. [66] with permission.

Solid dispersion technique is another method that is widely used for generating nanocapsulation systems for wrapping a core material (the payload) with functionality (e.g., phlorotannin) inside a coating material. This technology can not only mask or retain flavor and increase solubility of the payload, but also protects it from degradation by environmental factors, and controls its release at the target site [82]. In a recent report, Qi et al. [66] investigated the potential of using polyvinylpyrrolidone (PVP) as the coating to produce nanocapsulated PVP–phlorotannin complexes (Figure 4) with improved bioactivities and bioavailability. Different loading ratios of PVP vs. phlorotannin were investigated, and an optimum ratio of 8:1 (*w/w*) was established. As shown in Figure 5, the results indicated that the PVP–phlorotannin nanoparticles showed a slow and sustained kinetic release of phlorotannin in simulated gastrointestinal fluids; they were non-toxic to HaCaT keratinocytes (i.e., skin cells), and they could reduce the generation of endogenous reactive oxygen species (ROS). A PVP-based solid dispersion system would be a good choice for making better nano-sized delivery systems for phlorotannins in medicine, food, and cosmetics [66,83]. 

**Figure 6:**
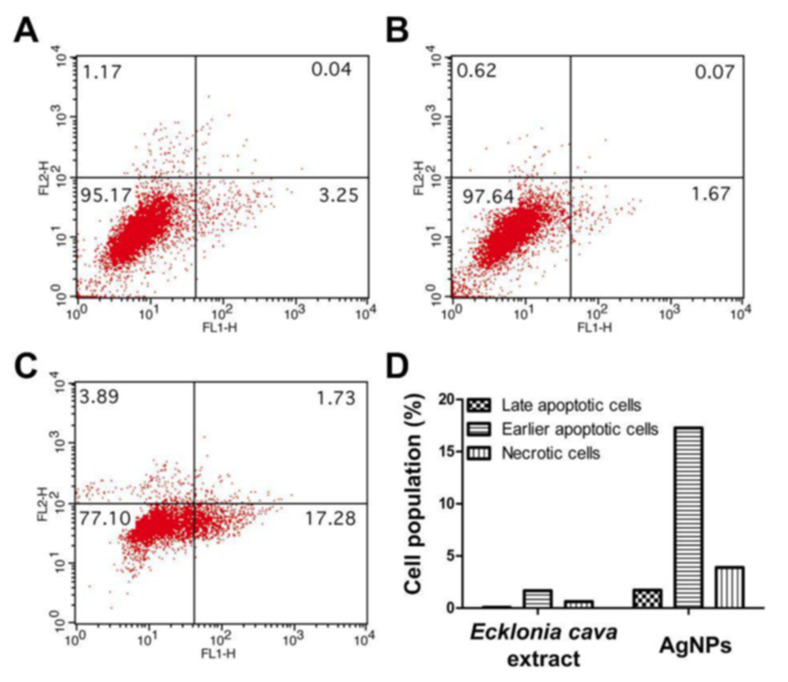
Annexin/PI staining of: (**A**) untreated HeLa cells; (**B**) HeLa cells treated with 250 μg/mL of E. cava extracts; (**C**) HeLa cells treated with 250 μg/mL of biosynthesized AgNPs; and (**D**) relative cell population of HeLa cells after treatment with *E. cava* extracts and biosynthesized AgNPs. Replicated from Shim et al. [69] with permission.

An interesting development with phlorotannin nano carriers was the synthesis of metallic nanoparticles (typically Au and Ag), with phlorotannins serving as the reducing agent. In these processes, the metallic nanoparticles formed have phlorotannin coating, hence can serve as delivery vehicles for phlorotannins (PhTs). Kim et al. [68] demonstrated that Au nanoparticles were formed within 1 min while mixing phlorotannins extract from *E. cava* with chloroauric acid at 80 °C, and the Au–PhT nanoparticles were 30 ± 0.25 nm and displayed promising antimicrobial activities. Machado et al [70] compared phlorotannins extracts from two macroalgae, *C. baccata* (CB) and *C. tamariscifolia* (CT), for their abilities to form gold nanoparticles; the results showed that CT possess three times more reducing power, almost four times more phenolic content, and four times more DPPH scavenging activity than CB, and the gold nanoparticles produced presented a non-cytotoxic profile in lower concentrations in mouse cell line L929 and human cell line BJ5-ta, which were efficient in cell regeneration, although with some differences between both species. The CT–gold nanoparticle complex had significantly better antioxidant bioactivity than the CB–gold nanoparticle complexes. Shim et al. [69] reported a green synthesis process for silver nanoparticles. The phlorotannin–silver nanoparticle complex has an average size of around 40 nm with spherical structure. These phlorotannin–silver nanoparticle complexes displayed antibacterial and antioxidant bioactivities. In particular, they exhibited a strong apoptotic anticancer activity against human cervical cancer cells that was not observed for phlorotannin, which suggested that the synergistic effects of phlorotannins and silver nanoparticles could further enhance their anticancer activities (Figure 6). Abedel-Raouf et al. [74] reported that phlorotannin isolated from *G. elongate* can be utilized to synthesize phlorotannin–gold nanoparticle complexes. Several shapes of phlorotannin–gold nanoparticles were formed, as shown by TEM. Triangular, truncated triangular, hexagonal, and rod shapes were detected. The sizes of these phlorotannin–gold nanoparticle complexes were around 3–77 nm. Antibacterial bioactivity was tested against *E. coli*, *K. pneumoniae*, and MRSA. Phlorotannin–gold nanoparticle complexes of 13 nm demonstrated the best antibacterial performance. It could be concluded that phlorotannin-induced Au and/or Ag nanoparticles could potentially become effective antibacterial, antiviral, and antioxidant reagents [75].

## 5. Conclusions

In this review, we summarized extraction techniques and bioactivities of phlorotannins and the strategies that have been explored via utilization of nano-sized delivery systems to improve their bioavailability and bioactivities. With nano-carriers protecting phlorotannins against GI tract biotransformation and degradation, phlorotannins can be released in a controlled manner to enhance their therapeutic functionalities, which included anticancer, anti-inflammatory, anti-HIV, antidiabetic, antioxidant, antibacterial activities, etc. This review serves as a roadmap for the development of more effective phlorotannin utilization strategies to fully take advantage of the tremendous potential of these algae-derived natural compounds as drugs and nutritional supplements for the pharmaceutical, food, and cosmetics industries.

## Figures and Tables

**Table 1:** Six basic types of phlorotannin in brown algae.

Type of Phlorotannin	Basic Linkage	Representative Structural Formula
Fucols	aryl–aryl linkages	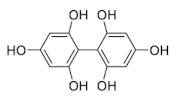
Fucophlorethols	aryl–aryl and aryl–ether	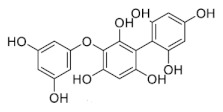
Fuhalols	aryl–ether linkages, OH groups in every third ring	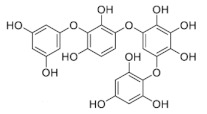
Carmalols	dibenzodioxin linkages	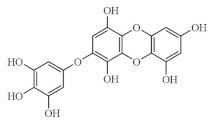
Phlorethols	aryl–ether linkages	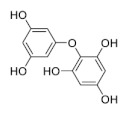
Eckols	a dibenzodioxin element substituted by a phenoxyl group at C-4	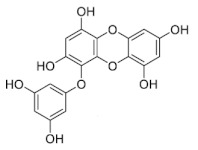

**Table 2:** Examples of reported anti-inflammatory effects of phlorotannins.

Compounds	Origin	Anti-Inflammation Effects	Test System	Dosage *	Reference
Phlorotannins extraction	*U. pinnatifida* sporophyll	Suppress H_2_O_2_-induced damage to cells.	RAW 264.7 cells	2.5–80 μg/mL	[8]
Phloroglucinol	*E. cava*	Suppress tumor necrosis factor-α, interleukin-1β, interleukin-6, and prostaglandin E2 produced by lipopolysaccharide.	HT1080 cells	1, 5, 10 μM.	[44]
		Suppress matrix metalloproteinase express to reduce chronic inflammation.	RAW264.7 cells	1, 5, 10 μM.	
Phloroglucinol and dieckol	*E. cava*	Suppress binding of IgE and FcɛRI.	KU812 cells	12.5, 25, 50, 100 μM	[111]
Phlorolfucofuroeckol	*E. stolonifera*	Suppress iNOS and COX-2 gene’s expression. Suppress cytokines in macrophages, which stimulate inflammatory activity. Suppress transcriptional activity of AP-1 and NF-kBs. Suppress AKt and P38 MAPK’s activation.	RAW 264.7 cells	20 μM	[45]

* Blank was used as negative control in all the cited research.

**Table 3:** Anti-HIV bioactivities of phloroglucinol and phlorotannins.

Compounds	Origin	Anti-HIV Effects	Test System	Dosage *	Reference
6,6′-bieckol	*E. cava*	Suppress HIV-1 (human immunodeficiency virus type 1) induced syncytia formation, production of vrial p24 antigen, effects of lytic.	C8166 and CEM-SS cells	0.5, 2.5, 5, 25, 50, 250, 500 μM	[11]
Arzanol (phloroglucinol α-pyrone)	*H. italicum* ssp. microphyllum	Suppress NF-κB and replication of HIV-1.	human T lymphocyte cell (Jurkat cell)	5, 10, 25 μM	[112]
Mallotophenone, mallotojaponin and mallotochromene	*M. japonicus*	Mallotophenone can suppress HIV-reverse transcriptase. Mallotophenone can suppress NF-κB.	(rA)n.(dT)12–18 as primers	10 μg/mL	[113]
Synthesized dimeric phloroglucinols	N/A	Suppress HIV-1 NL4.3 virus in vitro.	Human CD4+ T cell line (CEM-GFP)	20 μg/reaction	[114]
Prenylated phloroglucinols	*H. scruglii*	Suppress replication of HIV-1.	RDDP assay	3.5–8 μM	[115]

* Blank was used as negative control in all the cited research.

**Table 4:** Antidiabetes functionality of phlorotannins.

Compounds	Origin	Antidiabetic Activities	Test System	Dosage *	Reference
Dieckol	*E. cava*	Suppress α-glucosidase.	Recombinant Human Aldo-keto Reductase rhAKR1B10	10 μM	[57]
6,6′-Bieckol	*E. cava*	Suppress PTP 1B.	Same as above	10 μM	[57]
7-Phloroeckol	*E. cava*	Suppress ACE and α-glucosidase.	Same as above	10 μM	[57]
2-phloroeckol	*E. cava*	Suppresss α-glucosidase and PTP 1B.	Same as above and pNPP substrate	10 μM. 10 and 5 mM	[54,55,57]
			α-amylase, α-glucosidase, glucose induced protein glycation and glucose diffusion through dialysis membrane	133.33 µg/mL to 6.66 µg/mL	
Phlorofucofuroeckol-A	*E. cava*	Suppress α-glucosidase, PTP 1B, ACE, AGEs, and Aldose reductase.	pNPP substrate	25 and 10 mM	[54,55,56,58]
			AGE assay	200, 100, 50 mg/mL Positive contol: aminoguanidine	
			α-amylase, α-glucosidase, glucose-induced protein glycation and glucose diffusion through dialysis membrane	133.33 µg/mL to 6.66 µg/mL	
Phloroglucinol, Eckol, Dieckol, and Phlorofucofuroeckol	*E. stolonifera*	All can suppress α-glucosidase	Recombinant Human Aldo-keto Reductase rhAKR1B10 and type 2 diabetic db/db mice	10 μM	[53,57]

* Blank was used as negative control in all the cited research.

**Table 5:** Antioxidant effects (including photo-oxidative stresses) of phlorotannins.

Compounds	Origin	Antioxidant Effects	Test System	Dosage *	Reference
Dieckol	*E. cava*	Suppress UAB radiation induced photo-oxidative stress.	Human fibroblaste cell	5, 50, 250 μM.	[59]
		Suppress UV-B radiation induced cell damages (both DNA damage and nuclear fragmentation).	Human fibroblaste cell	5, 50, 250 μM.	
Diphlorethohydroxycarmalol (DPHC)	Brown algae.	DPHC can scavenge UV-B radiation induced ROS; with more DPHC added into the treatment, cell viability was uplifted.	Human fibroblast cells	5, 50, 250 μM.	[62]
Dieckol	*E. cava*	Suppress cell damage induced by UV-B radiation in vitro. Suppress the level of ROS, nitric oxide (NO) and cell death all stimulated by UV-B radiation.	HaCaT cells	5, 50, 100, and 250 μM.	[63]
Phlorotannin extract (PE)	*E. cava*	Suppress levels of ROS and NO induced by UV-B radiation. In addition, cell death rate can be reduced by pre-treating zebra fish embryos with PE.	Zebra fish	5, 50, 100, and 250 μM.	[63]
Diphlorethohydroxycarmalol (DPHC)	*I. okamurae*	Suppress high glucose-induced oxidative stress. Suppress level of ROS (reactive oxygen species), NO (nitric oxide) induced by high glucose.	Human umbilical vein endothelial cells	5, 25, and 50 μM.	[105,118]
		Suppress high glucose induced inducible nitric oxide synthase (iNOS), cyclooxygenase-2 (COX-2), and activation of nuclear factor-kappa B (NF-κB) activation.	Human umbilical vein endothelial cells	5, 25, and 50 μM.	

* Blank was used as negative control in all the cited research.

## Data Availability

Not applicable.
